# Phytochemical Analysis and Anti-Inflammatory Potential of *Acanthus mollis* L. Rhizome Hexane Extract

**DOI:** 10.3390/ph16020159

**Published:** 2023-01-22

**Authors:** Nuria Acero, Dolores Muñoz-Mingarro, Inmaculada Navarro, Antonio J. León-González, Carmen Martín-Cordero

**Affiliations:** 1 Pharmaceutical and Health Sciences Department, San Pablo-CEU University, CEU Universities, Urb. Montepríncipe, 28668 Madrid, Spain; 2 Chemistry and Biochemistry Department, San Pablo-CEU University, CEU Universities, Urb. Montepríncipe, 28668 Madrid, Spain; 3 Department of Physical Chemistry, Faculty of Pharmacy, University of Seville, C/P. García González, 2, 41012 Seville, Spain; 4 Department of Pharmacology, Faculty of Pharmacy, University of Seville, C/P. García González, 2, 41012 Seville, Spain

**Keywords:** *Acanthus mollis*, Acanthaceae, anti-inflammatory activity, phytosterols

## Abstract

The rhizomes of *Acanthus mollis* have traditionally been used for the treatment of several ailments involving inflammation. However, to the best of our knowledge, their chemical composition and pharmacological properties have not been studied until now. As a first approach, this study analyses the *A. mollis* rhizome hexane extract phytochemistry and its anti-inflammatory and antioxidant capacities in HepG2 and RAW 264.7 cell culture assays. Chemical profiling was performed with gas chromatography mass spectrometry without the modification of native molecules. Free phytosterols (such as β-sitosterol) account for 70% of detected compounds. The anti-inflammatory capacity of the rhizome extract of *A. mollis* is mediated by the decrease in the NO production in RAW 264.7 that has previously been stimulated with lipopolysaccharide in a dose-dependent manner. Furthermore, HepG2 pre-treatment with the rhizome extract prevents any damage being caused by oxidative stress, both through ROS scavenge and through the antioxidant cellular enzyme system. In this respect, the extract reduced the activity of glutathione peroxidase and reductase, which were stimulated under oxidative stress conditions. Our results suggest that the extract from the rhizomes of *A. mollis* may constitute a potential source of natural products with anti-inflammatory activity and could validate the traditional use of *A. mollis.*

## 1. Introduction

Inflammation is a defence mechanism in response to harmful stimuli, such as pathogens, and to tissue damage. It is involved in many pathologies, such as allergies, asthma, and hepatitis, and in autoimmune diseases, such as celiac disease and rheumatoid arthritis. It is also related in its chronic form with diabetes, osteoarthritis, cardiovascular diseases, obesity, and cancer [1]. The inflammation with which these pathologies occur is associated with an excess of free radicals, which causes oxidative damage and plays a crucial role in the development and maintenance of this process [2,3]. Furthermore, inflammation reduces cellular antioxidant capacity [3]. Therefore, the two processes of inflammation and oxidative stress are closely related. The available arsenal of anti-inflammatory drugs includes non-steroidal anti-inflammatory drugs, corticosteroids, and immunosuppressant drugs. However, there is a growing interest in the search for new anti-inflammatory remedies given the lack of effectiveness in certain cases and the side effects of the current therapies. The number of new plant-derived anti-inflammatory compounds is continuously rising. Polyphenols, such as quercetin, epigallocatechin-3-gallate, curcumin, and resveratrol, and alkaloids such as colchicine and capsaicin have shown this pharmacological ability in clinical studies [4]. On the other hand, from among those substances obtained from plants, phytosterols also deserve mention. Molecules, such as sitosterol, stigmasterol, and campesterol, have a known lipid-lowering effect by decreasing the absorption of cholesterol [5], but they have also been shown to possess anti-inflammatory properties [6]. Therefore, β-sitosterol can be used in pathologies such as benign prostatic hyperplasia, colon and breast cancer, atherosclerosis, and gastrointestinal ulcers [7].

Acanthaceae is considered one of the leading families of the dicotyledonous flowering plants. This family consists of 242 genera and 3947 species, mainly distributed across tropical and subtropical zones, primarily in South and Central America, Africa, Southern Asia, and Australasia. However, some species are also located in temperate areas of the Mediterranean basin [8]. The *Acanthus* genus, with 32 species, comprises perennial herbs and rarely small subshrubs. They have basal leaves and flowers on terminal spikes with spiny bracts. The latter characteristic gives the genus its name which derives from the Greek word for spiny. As occurs in other botanical genera, certain *Acanthus* species have been used in traditional medicine. From among these, the only species native to Europe is *Acanthus mollis* L. This plant has been held in high esteem since ancient times when it was used by architects and artists in ornamental designs, as shown by its incorporation as a characteristic decorative element in Corinthian columns. This herbaceous plant with rhizomes presents shiny, dark green, deeply dissected basal leaves, and its flowers bloom in late spring to midsummer. Each flower is subtended by a spiny bract. The flowers have a large, purple to green upper calyx-lobe and fused white petals [9]. Commonly named bear’s breeches, this plant has been traditionally used for the treatment of several ailments. In Sicily, aerial parts are indicated in the treatment of psoriasis. Moreover, in Italy, Greece, Spain, and Madeira, its leaves are employed as a vulnerary for skin diseases, burns, and wounds. Its leaves are employed in the treatment for inflammation of mucous membranes in the respiratory and digestive tracts, for intestinal disorders, diarrhoea, colitis, haemoptysis, toothaches, and stomatitis. Its leaves are employed for headaches, convulsions, nervousness, rheumatism, contusions, and swollen legs and feet. They are also used as a diuretic and analgesic for the urinary tract [10,11,12,13,14,15,16,17,18,19,20]. To date, several studies have been conducted to analyse the pharmacological properties and the phytochemical composition of the aerial parts of this plant [21]. The anti-inflammatory and anti-hemorrhoidal capacity of its leaves and their antifungal and antioxidant abilities have been reported [22,23]. Furthermore, the presence of phenylpropanoids, benzoxazinoids, flavonoids, and verbascoside have been identified [17]. This last compound, isolated from *A. mollis* leaves, has demonstrated neuroprotective activity [24]. Although the leaves of this plant have been studied both in terms of their chemical composition and with respect to their pharmacological properties, to the best of our knowledge, their rhizomes have not been studied until now. The aim of the present study was to analyse the *A. mollis* rhizome hexane extract phytochemistry and its anti-inflammatory and antioxidant capacity in cell culture assays. 

## 2. Results

### 2.1. Chemical Profiling of A. mollis Hexane Extract 

**Figure 1:**
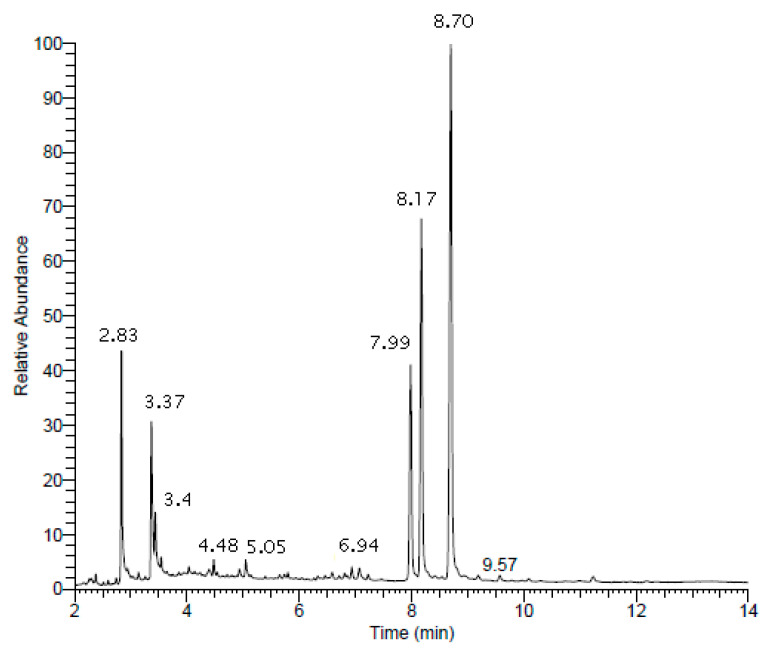
GC-MS chromatographic profile of the hexane extract of *A. mollis* rhizomes.

The hexane extract was characterised with gas chromatography mass spectrometry (GC-MS) without modification of the native molecules (Figure 1).

**Figure 2:**
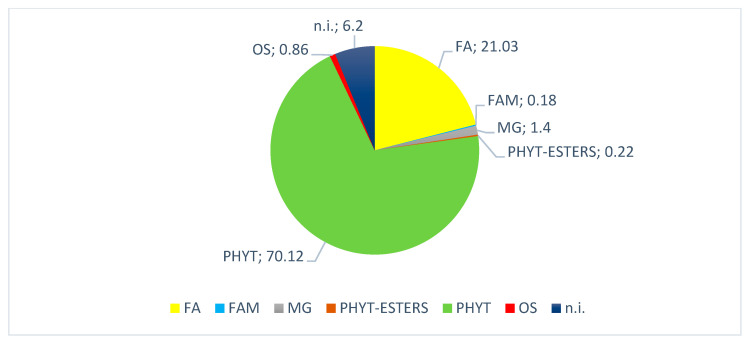
The mean relative content of the main chemical classes for hexane extract according to features identified in the GC-MS dataset. FA: free fatty acids; FAM: fatty acid methyl esters; MG: monoglycerides; PHYT-ESTERS: phytosterol esters; PHYT: free phytosterols; OS: other steroids; n.i.: not identified.

This analysis enabled the identification of sixteen known compounds (Table 1) of different chemical classes and provided a comprehensive overview of the main chemical content of hexane extract. The majority of the compounds detected were free phytosterols and accounted for 70% of the mean total ion chromatogram (TIC) area. Among these phytosterols, β-sitosterol was found to be the most abundant sterol (35.4%). Fatty acids accounted for 21%, and the remaining parts belonged to unidentified features and other chemical classes. A small amount (0.2%) was annotated as phytosterol esters (Figure 2).

### 2.2. Extract and β-Sitosterol Cytotoxicity 

No significant cytotoxicity was detected in the macrophages, nor in the hepatocarcinoma cell lines at concentrations of up to 100 µg/mL of A. mollis rhizome hexane extract or β-sitosterol.

### 2.3. Inhibition of Nitric Oxide Production by Macrophages

**Figure 3:**
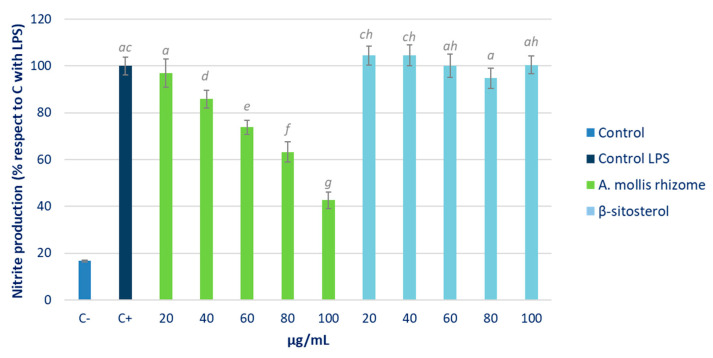
Percentage of NO with respect to the positive control stimulated with LPS (C+) in samples treated with various concentrations of *Acanthus* rhizome and β-sitosterol. Different letters indicate significant differences (ANOVA-Test, Bonferroni *p* < 0.05).

In order to analyse the anti-inflammatory capacity of the *Acanthus* rhizome extract, a change in NO production in a RAW 264.7 cell culture subsequent to being stimulated with LPS was determined. The results are shown in Figure 3 as the percentage of NO production with respect to the positive control (cells stimulated with LPS and treated with PBS). β-sitosterol was used as the reference compound. 

A dose-dependent decrease in NO production was observed in cells treated with the rhizome extract. At the same concentrations, β-sitosterol showed no effect, while *Acanthus* extracts, even at low doses, induced a significant reduction in NO production. As can be observed, the NO decrease at the highest rhizome concentration (100 μg/mL) reached levels close to 60% with respect to the production of LPS-induced control cells. 

### 2.4. Intracellular Reactive Oxygen Species Measurement

The assay was performed in a HepG2 cell culture growing both under normal culture conditions and also after oxidative stress induction with hydrogen peroxide. The HepG2 cell line of human hepatocarcinoma, which reproduces hepatocytes, was taken as a model that has been widely used to evaluate the effects of different compounds of natural origin in vitro [25].

**Figure 4:**
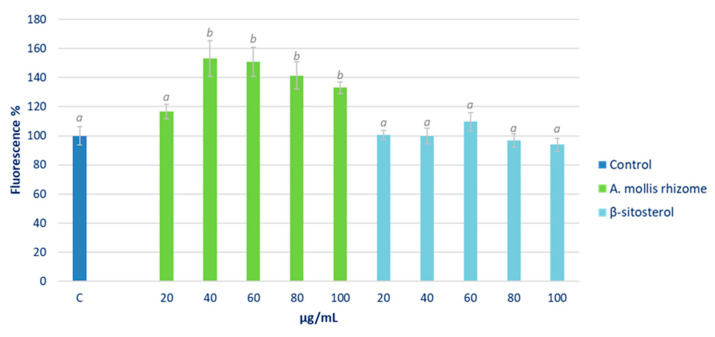
Effect of different concentrations of *Acanthus* rhizome extract and β-sitosterol on ROS levels in a HepG2 cell culture growing under normal culture conditions. Cells were treated with 0 (control, C), 20, 40, 60, 80, and 100 μg/mL of extract or reference substance for 90 min. Different letters indicate significant differences (ANOVA-Test, Bonferroni *p* < 0.05).

The percentage of ROS related to the control after 90 min of treatment in cells growing under normal culture conditions is shown in Figure 4.

The highest rhizome extract concentrations tested produced a significant increase in fluorescence, and therefore in intracellular ROS levels, while no effect was detected in cells under β-sitosterol treatment.

**Figure 5:**
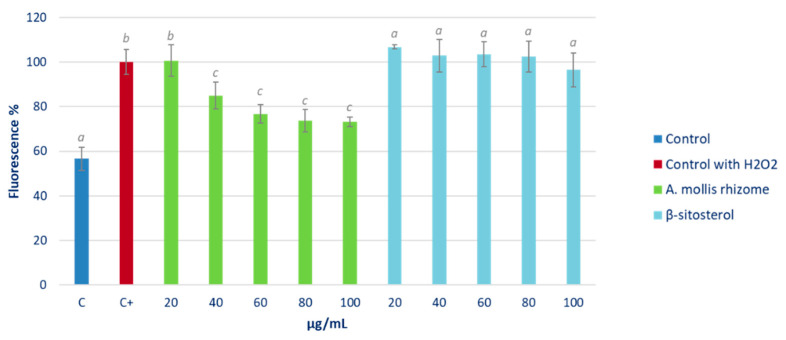
Effect of various concentrations of *Acanthus* rhizome extract and β-sitosterol on ROS levels in a HepG2 cell culture after 90 min of inducing oxidative stress with H_2_O_2_ 200 mM. Cells were pre-treated with 0 (control, C), 20, 40, 60, 80 and 100 μg/mL of extract or reference substance for 24 h before inducing oxidative stress. Different letters indicate significant differences (ANOVA-Test, Bonferroni *p* < 0.05).

Furthermore, the evaluation of whether pre-treatment with the hexane extract or with β-sitosterol limited the production of ROS under induced oxidative stress was also analysed in a HepG2 cell culture. The results are shown in Figure 5.

It can be observed that the pre-treatment with the four highest rhizome extract concentrations significantly reduced the production of ROS in a dose-dependent manner. On the other hand, β-sitosterol, only at the highest concentration, was able to reduce ROS levels, albeit not significantly. This compound did not have the capacity, at the tested concentrations, to decrease the concentration of ROS inside the cell under oxidative stress. 

### 2.5. Antioxidant Enzyme Assay

**Figure 6:**
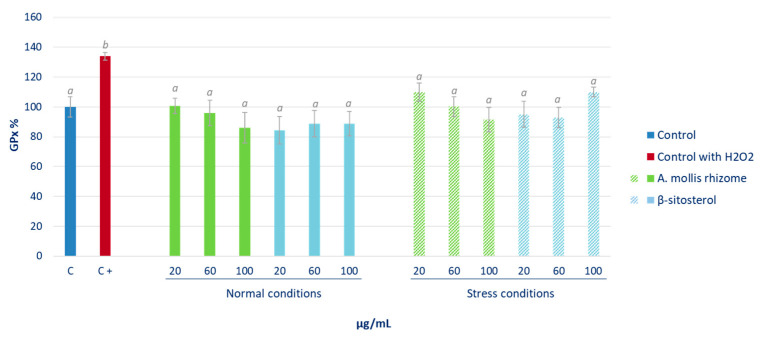
Effect of *Acanthus* rhizome extract and β-sitosterol (μg/mL) on glutathione peroxidase (GPx) activity. The percentage of GPx activity with respect to non-stressed control cells is represented. The results are expressed as mean ± SD (*n* = 3). Different letters indicate significant differences (ANOVA-Test, Bonferroni *p* < 0.05).

An evaluation was carried out to analyse the effect of *Acanthus* rhizome extract and β-sitosterol on the activity of the cellular antioxidant enzyme system: glutathione peroxidase (GPx) and glutathione reductase (GR). As in the previous assay, cells growing under normal conditions and under induced oxidative stress were both analysed. The effects of the phytoextract and the reference compound on GPx activity are shown in Figure 6. Related to cells growing under normal conditions, no effect was detected between treatment with control cells and rhizomes or β-sitosterol. With *Acanthus* extracts, the activity of GPx decreased in a dose-dependent manner, although not significantly, while no effect was observed when cells were treated with β-sitosterol. Under induced oxidative stress, both treatments were able to reverse the effect of oxidative stress and recover the baseline levels of activity of this enzyme. 

**Figure 7:**
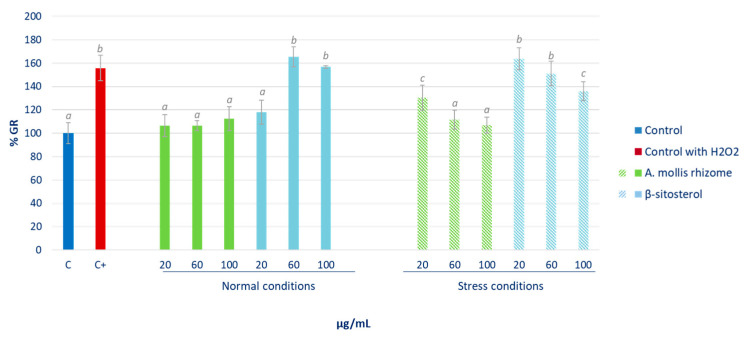
Effect of *Acanthus* rhizome extract and β-sitosterol (μg/mL) on glutathione reductase (GR) activity. The percentage of GR activity with respect to non-stressed control cells (C) is represented. The results are expressed as mean ± SD (*n* = 3). Different letters indicate significant differences (ANOVA-Test, Bonferroni *p* < 0.05).

Glutathione reductase (GR) is also a key enzyme within the endogenous antioxidant system. In the present study, the activity of GR varied following a less clear trend than did the variation observed in GPx activity. As can be observed in Figure 7, the rhizome extract did not affect GR activity in cells under normal conditions. While under stress conditions, as in the previous case, it recovered non-stressed control levels at higher doses. β-sitosterol treatment yielded clearly different results. Related to normal growing conditions, the higher doses of 60 and 100 μg/mL of this compound were able to significantly increase the activity of GR. However, the pre-treatment of the cells with all the concentrations tested remained unable to reduce this activity to normal levels, although the 100 µg/mL dose was able to reduce the increase caused by the oxidative stress. 

## 3. Discussion

The aim of the present study was to assess the phytochemical composition and anti-inflammatory activity of a hexane extract obtained from *A. mollis* rhizomes in an ultrasonic bath at 50 °C for 1 h. These rhizomes constitute an abundant raw material as this plant is commonly used for ornamental purposes in Europe and has been revealed to be an invasive species that can aggressively spread in optimum conditions. 

GC-MS without the modification of native molecules led to the identification of sixteen known, mainly lipophilic, compounds (Table 1). The presence of free phytosterols, and more specifically that of β-sitosterol, should be highlighted (Figure 2). However, other compounds, such as phytosterol esters, may be of great interest given their different bioavailability with respect to free forms [26].

Nitric oxide (NO) regulates the processes of neuronal communication, vasodilation, and neurotoxicity. However, it is also a pro-inflammatory mediator [27] and its overproduction induces tissue damage associated with this process. Nitric oxide is produced by macrophages, and the control of its production is essential to reduce inflammation. Macrophages are activated by lipopolysaccharides (LPSs), components of the cell wall of gram-negative bacteria, which induce the production of pro-inflammatory mediators such as NO and prostaglandins, pro-inflammatory cytokines such as IL-6, IL-1β, and TNF-α, and enzymes such as inducible nitric oxide synthase (iNOS) and cyclooxygenase-2 (COX-2) [28]. 

Figure 3 shows the effect of the β-sitosterol and *Acanthus* hexane extracts over NO production in an LPS-stimulated RAW 264.7 cell culture. No effect was detected after the reference compound treatment. β-sitosterol has demonstrated potent anti-inflammatory activity in vivo in studies on specific (immune reaction) and non-specific acute inflammation carried out on rodents [7]. Specifically, β-sitosterol controls the production of inflammatory mediators acting through ERK, p38, and NF-κB pathway inhibition [29]. The LPSs employed to stimulate macrophages are capable of activating TLR4 receptors. These receptors activate kinases, the activity of which results in the release of NF-κB p50/p65 dimers in the cytoplasm. Once NF-κB is translocated to the nucleus, it binds to the DNA and regulates the expression of inflammatory cytokines such as IL-1β, IL-6, TNF-α, IL-12, and INF-β. β-sitosterol inhibits the activation of NF-κB by downregulating the expression of TLR4, and consequently also of those cytokines mentioned above [30]. The anti-inflammatory capacity of β-sitosterol is dose-dependent and similar to that achieved by ibuprofen and even by prednisone. Likewise, its ability to reduce edema and the secretion of pro-inflammatory cytokines, such as TNF-α, has been proven in animal studies by increasing, in contrast, the production of anti-inflammatory cytokines. IL-10 is an anti-inflammatory cytokine which acts as a regulatory factor that could potentially suppress the TNF-α, IL-1β, and IL-8 cascades [31]. Liu et al. [32] suggested the pivotal role of IL-10 in the β-sitosterol-induced anti-inflammatory response. β-sitosterol can induce an increase in these cytokine levels in both in vitro and in vivo assays. Our results suggested that the anti-inflammatory mechanism of action of β-sitosterol was not involved in the reduction of NO levels. Boukes and van de Venter [33] observed similar results in the culture of the U937 cell line of human promonocytic leukaemia. However, the *A. mollis* rhizome extract offered promising results in this regard, as it was able to reduce the NO production in a dose-dependent manner. In order to confirm the anti-inflammatory capacity of the extract, its effect on pro-inflammatory cytokine levels should be analysed in future studies. 

Reactive oxygen species (ROS) are molecular signals that are implied in normal physiological processes. However, in excess, they cause cytotoxic effects, which have been extensively documented [24]. Therefore, since cell oxidative stress is directly related to inflammation, the effect of the *A. mollis* extract and β-sitosterol on intracellular ROS levels was analysed. In this respect, the HepG2 cell treatment with β-sitosterol showed no effects, while the highest tested rhizome extract concentrations produced a significant increase in intracellular ROS levels (Figure 4). Boukes and van de Venter [33] also observed an increase in intracellular ROS levels in monocytes and monocyte-d/macrophages U937 treated with a *Hypoxis* spp. extract rich in phytosterols. The authors related these results to a pro-inflammatory response. However, the increase produced by the *Hypoxis* extract attained up to 500%, while in this study, the increase reached only 153.2%. It should be borne in mind that, in practice, a moderate pro-oxidant effect may raise the antioxidant defences of the cell, which would lead to greater protection against oxidative stress. In order to ascertain whether the observed increase in ROS levels could be considered mild or moderate and therefore positive for the cell, we analysed the effect of the extract in cells growing under induced oxidative stress. Under these conditions, pre-treatment with the hexane extract significantly reduced the intracellular oxidative stress; this effect was more marked when higher concentrations were used (Figure 5). Additionally, only the highest tested concentrations of β-sitosterol showed antioxidant effects in this assay. Our results were consistent with those obtained by Boukes and van de Venter [33] who did not find any effect of β-sitosterol on ROS levels in human promonocytic leukaemia U937 cells. On the other hand, Moreno [34] reported a reduction in the superoxide anion (O_2_^−^) and H_2_O_2_ levels in the RAW 264.7 cell line stimulated with PMA (phorbol-12-miristate-13-acetate) after treatment with β-sitosterol at concentrations equivalent to those used in this study. The author concluded that this ability of β-sitosterol was not due to its ability to capture free radicals, but instead to its capacity to act on the intracellular antioxidant system, and hence its results in this respect were in concordance with those obtained in this study.

The greater effect of the extract compared to the reference substance (Figure 5) could be due to either a synergistic effect of its components or to the presence of phytosterol esters. In the human colon adenocarcinoma cell line HT-29, β-sitosterol ferulate has demonstrated the ability to reduce intracellular ROS levels similar to those of α-tocopherol and N-acetylcysteine [35]. Phytosterol esters appear to exhibit greater bioavailability, which improves their bioactivity. 

The uncontrolled production of ROS that takes place during oxidative stress contributes to the pathogenesis of cardiovascular disease, cancer, and inflammation. High levels of ROS are involved in the activation of the arachidonic acid signalling pathway [36], and hence, the hexane extract of *A. mollis* rhizomes could show an anti-inflammatory effect through this antioxidant mechanism of action.

GPx activity catalyses the reduction of peroxide radicals to alcohols and oxygen, and of H_2_O_2_ to water and oxygen, thereby limiting its harmful effect on cells [37]. Therefore, the activity of this enzyme plays a fundamental role in protecting against oxidative stress.

The presence of H_2_O_2_ induced a significant increase in GPx enzyme activity, thereby indicating a clear response of the cell’s antioxidant system to oxidative stress, as can be deduced by comparing the two controls (C of non-stressed cells, in blue; and C+ of stressed cells, in red) (Figure 5). Both the extract and β-sitosterol were able to reverse the effect of oxidative stress after H_2_O_2_ treatment. In view of these results, the presence of phytosterols in the hexane extract of the rhizomes could explain this activity, being able to recover baseline levels of this enzyme activity. 

These GPx and GR enzymes are vital mechanisms in the cellular defence against oxidative damage. Changes in the activity of these antioxidant enzymes can be considered as biomarkers of the antioxidant response [38]. Glutathione peroxidase catalyses GSH oxidation into oxidised glutathione at the expense of H_2_O_2_ or other peroxides, and GR recycles oxidised glutathione back to GSH [39]. For this reason, it can be observed how the induction of oxidative stress in HepG2 cells with H_2_O_2_ leads to the increase in the activity of these enzymes in order to reduce the levels of reactive species therein. However, a rapid return to the baseline values of activity of these proteins once the stress is overcome places the cell in a favourable condition to face a new aggression [40]. Therefore, the ability to reduce the levels of the activity of GPx of both the extract and the β-sitosterol at all tested contractions under the conditions of oxidative stress granted them a notable protective effect against this type of alteration. In view of the results, it can be concluded that certain bioactive compounds present in the extract, such as β-sitosterol, did not alter the basal status of the cell, but they did protect it through the endogenous antioxidant system in situations of oxidative or inflammatory stress, as reported by other authors for yerba mate and green coffee extracts [41]. 

## 4. Materials and Methods

### 4.1. Experimental Procedures

All chemicals were of analytical reagent grade purchased from Sigma (Merck KGaA, Darmstadt, Germany). Glutathione reductase from baker’s yeast (*S. cerevisiae*) was purchased from Sigma-Aldrich (Madrid, Spain). Proteins were quantified using a BCA protein assay kit from Thermo Scientific (Gul Cir, Singapore). Fluorescence was measured in a Fluostar optima plate reader (BMG Labtech, Offenburg, Germany), at an excitation wavelength of 485 nm and 529 nm of emission.

### 4.2. Plant Material and Extract Preparation

*Acanthus mollis* rhizomes were collected in their plant vegetative stage in September 2017 in La Isla, Colunga (Asturias, Spain) (43°48′39.5″ N–5°22′48.13″ W). All sampled plants were over 10 years old. Plant material was identified and authenticated by the Botanical Department of San Pablo–CEU University in Madrid. A voucher specimen (Ref. 3372) was deposited into the Faculty of Pharmacy Herbarium, San Pablo–CEU University in Madrid. The major development of leaves and rhizomes, together with the long photoperiod and high intensity of light, made September an appropriate period for the collection of rhizomes [42]. The rhizomes were dried at room temperature (20–25 °C) and then spread evenly and turned over every 24 h. When the humidity was reduced to values lower than 10–12%, the rhizomes were ground into powder using a Culatti grinder equipped with a 1 mm link filter (Janke and Kunkel GMGH, Staufen, Germany). The ground material was extracted three times with hexane in an ultrasonic bath (Ultrasons, Selecta^®^, Barcelona, Spain) at 50 °C for 1 h in a solvent-to-solid ratio of 10mL/g. Hexane was chosen as a solvent due to its attributes, such as a simple recovery, non-polar nature, and low latent heat of vaporisation. This enabled the extraction of non-polar molecules of great pharmacological interest [43]. The extract was filtered through Whatman no. 1 filter paper and subsequently evaporated to dryness under a reduced pressure at <50 °C using a Buchi rotary evaporator. The concentrated extract was then dried to a constant weight under a stream of cold air at room temperature (22 °C) and stored at 4 °C until use. The extract yield was 0.37% (*w*/*w*). 

### 4.3. Cell Culture

The HepG2 human hepatocarcinoma and the RAW 264.7 murine macrophage cell lines were obtained from the European Collection of Cell Cultures (Health Protection Agency, London, UK) (ECACC ref. 85011430 and ref. 91062702, respectively).

In both cases, cells were grown in EMEM (Eagle’s Minimum Essential Medium) supplemented with 2 mM glutamine, 1% non-essential amino acids, 10% foetal bovine serum (FBS), and 1% antibiotics (10 mg/mL streptomycin and 10,000 U penicillin). They were kept in an incubator at 37 °C in an atmosphere of 5% CO_2_ in air. The state of the culture was regularly checked every 24 h by observation under a microscope.

### 4.4. GC–MS Analysis 

An analysis of the hexane extract was carried out using a Thermo Trace 1300 gas chromatography equipped and coupled to a mass detector Thermo TSQ 8000 spectrometer with a ZB-5ms (30 m × 0.25 mm, 0.25 μm) capillary column. The hexane extract was dissolved in n-hexane and directly injected in GC-MS [44]. The samples were injected at 310 °C in split mode (split ratio 25:1) with a volume of 1.0 μL. Helium was utilised as the carrier gas at a constant flow rate of 1 mL/min. The GC temperature program was initiated at 200 °C and held for 0.5 min, increased to 310 °C at 30 °C/min and held for 10 min. An electron impact ionisation (EI) source was applied, the electron energy was 70 eV, the source temperature was set at 280 °C, and the interface temperature was set at 310 °C. The compounds were determined through standard compounds, mass spectra in a database (NIST 12.0) (National Institute of Standards and Technology), and the chromatographic data (retention indices) reported in the literature [44].

### 4.5. Cytotoxicity Assay 

The cytotoxic effect of the extract was evaluated by an MTT assay. The cells were exposed to a variety of extract and β-sitosterol concentrations for 24 h (20, 40, 60, 80, and 100 μg/mL diluted in phosphate-buffered saline (PBS)). The concentrations tested were serial dilutions of a DMSO stock solution with PBS. Final dimethyl sulfoxide (DMSO) concentrations in the cell assays remained below 0.1%. These concentrations of DMSO did not affect cell viability. Phosphate-buffered saline was used as a negative control and doxorubicin was used as a positive control [45]. The tests were conducted in triplicate.

### 4.6. Nitrite Production in RAW 264.7 with Griess Reagent

In order to determine the anti-inflammatory activity of the rhizome hexane extract, the inhibition of nitric oxide production was evaluated in RAW 264.7 cells (murine macrophage cell line) that had previously been stimulated with LPSs. This assay is based on the Griess reaction. The cells were seeded in a 96-well plate (6 × 10^5^ cells per well). The plate was kept in an incubator at 37 °C in an atmosphere with 5% CO_2_ for 24 h. The cells were then treated either with a culture medium (negative control) or with 1 μg/mL of LPSs either in the absence (positive control) or presence of various concentrations of *Acanthus* extract or β-sitosterol (20, 40, 60, 80, and 100 μg/mL) for 24 h. Subsequently, 100 μL of culture media was taken from each well and 90 μL of 1% sulphanilamide in 5% phosphoric acid in H_2_O was added. After 5 min at room temperature, protected from light, 90 μL of 0.1% N-(1-naphthyl)-ethylenediamine was added. The plate was incubated in darkness for 30 min, after which the absorbance was measured at 550 nm in a plate reader (Spectrostar Nano BMG Labtech, Ortenberg, Germany) [17]. All tests were conducted in triplicate.

### 4.7. Intracellular ROS Assay

In order to study the effect of *A. mollis* rhizome hexane extract against the production of ROS in a HepG2 cell culture, two growing conditions were evaluated: cells treated with the extract under normal culture conditions, and pre-incubation with the extract before oxidative stress was induced with 200 mM H_2_O_2_. To this end, 15,000 cells were seeded in each well of a 96-well plate in 200 µL of EMEM supplemented with 2 mM glutamine, 1% non-essential amino acids, 1% FBS, and 1% antibiotics. To carry out this assay, the percentage of serum in the medium was reduced in order to prevent any interaction between the compounds present in the extract and certain components of the serum that could otherwise trigger artifacts with cytotoxic activity [46].

For the assay under normal culture conditions, after 24 h of incubation, the medium was replaced by various concentrations of the extract or β-sitosterol dissolved in the corresponding culture medium supplemented with 1% FBS. Plates were incubated for a further 24 h at 37 °C in a 5% CO_2_ atmosphere. A 2-7-dichlorodihydrofluorescein (DCFH-DA) diacetate assay was then performed on the HepG2 cell line to determine intracellular ROS levels [24]. The measurement of fluorescence was started when the extract or the reference compound was added every 15 min up to a maximum of 90 min. Fluorescence was measured at an excitation wavelength of 485 nm and 529 nm of emission. The results were expressed as a percentage of fluorescence with respect to the control. 

For the study under oxidative stress, after 24 h of incubation, the medium was replaced by various concentrations of the extract or β-sitosterol dissolved in the corresponding culture medium supplemented with 1% FBS. After 24 h, the medium was replaced by 0.02 mM DCFH-DA for 30 min. The cells were then washed with PBS, and oxidative stress was induced with 200 mM of hydrogen peroxide. The measurement of fluorescence began when H_2_O_2_ was added every 15 min up to a maximum of 90 min. All tests were conducted in triplicate.

### 4.8. Measurement of Antioxidant Enzyme Activity

The effect of the *Acanthus* rhizome hexane extract or β-sitosterol on the activity of GPx and GR in the HepG2 cell line was evaluated. As in the previous assay, this effect was evaluated both under normal culture conditions and under conditions of oxidative stress.

In Petri dishes of 100 × 20 mm with 7 mL of culture media, 6 × 10^6^ cells were seeded. After 24 h of incubation, the medium was replaced with various concentrations of *Acanthus* rhizome extract or β-sitosterol dissolved in a culture medium supplemented with 1% FBS. 

For the assay under normal culture conditions, after 24 h of pre-treatment, the cells were collected with a scraper and lysed. For the study of cells under oxidative stress, after 24 h of pre-treatment, the medium was replaced with 7 mL of media in the case of control or with a medium with 200 mM H_2_O_2_, and the cells were incubated for 3 h. The cells were then collected and lysed.

The measurement of GPx activity was based on the GSH oxidation mediated by the enzyme coupled to the disappearance of NADPH as a cofactor of GR following the protocol of León-González et al. [40]. The GR activity was determined through the decrease in absorbance that occurred due to NADPH oxidation [47]. All tests were conducted in triplicate.

### 4.9. Statistical Analysis

A statistical data analysis was carried out through one-factor ANOVA after having verified the homogeneity of the variances using the Levene test under the IBM SPSS Statistics 24 program. Post hoc comparisons were performed by employing the Bonferroni test (*p* < 0.05). 

## 5. Conclusions

The anti-inflammatory capacity of the rhizome extract of *A. mollis* was mediated by a decrease in NO production and by the prevention of damage caused by oxidative stress, both through the scavenge of ROS and through the antioxidant cellular enzyme system. Our results suggested that the hexane extract from the rhizomes of *A. mollis* may constitute a potential source of natural products with anti-inflammatory activity. The isolation and purification of other active principles from this plant are now in progress, together with further studies to determine other mechanisms involved in their anti-inflammatory action.

## Figures and Tables

**Table 1:** Chemical profile of n-hexane extract from *A. mollis* L. rhizomes.

CLASS	Compounds	RT (min)	Area (%)
**Fatty acids methyl ester**	Palmitic acid, methyl ester	2.74	0.18
**Free fatty acids**	Palmitic acid	2.83	9.76
	Margaric acid	3.14	0.21
	Linoleic acid	3.36	7.62
	Stearic acid	3.44	3.44
**Monoglycerides**	Palmitin, 2-monoglyceride	4.48	0.59
	Linolein, 2-monoglyceride	5.05	0.81
**Phytosterol esters**	Campesteryl ester	6.46	0.11
	Sitosteryl ester	6.85	0.11
	Stigmasteryl ester	6.94	0.47
**Free phytosterols**	Cholesterol	7.23	0.22
	Campesterol	7.99	13.04
	Stigmasterol	8.17	21.48
	β-sitosterol	8.70	35.38
	Stigmasta-3,5-diene	7.07	0.59
	α-saccharostenone	9.57	0.27

## Data Availability

Data is contained within the article.
